# Ionic Liquid-Based Vacuum Microwave-Assisted Extraction Followed by Macroporous Resin Enrichment for the Separation of the Three Glycosides Salicin, Hyperin and Rutin from *Populus* Bark

**DOI:** 10.3390/molecules19079689

**Published:** 2014-07-07

**Authors:** Fengli Chen, Kailin Mo, Zhaizhi Liu, Fengjian Yang, Kexin Hou, Shuangyang Li, Yuangang Zu, Lei Yang

**Affiliations:** 1 Key Laboratory of Forest Plant Ecology, Ministry of Education, Northeast Forestry University, Harbin 150040, China; E-Mails: chenfengli1103@163.com (F.C.); zaizhiliu@hotmail.com (Z.L.); lynnhkx@hotmail.com (K.H.); Rachel.0527@hotmail.com (S.L.); zygorl@163.com (Y.Z.); 2 Sichuan Academy of Forestry, Chengdu 610081, China; E-Mail: mokailin@126.com

**Keywords:** ionic liquid vacuum microwave-assisted extraction (ILVMAE), *Populus* bark, macroporous resin, glycoside

## Abstract

An effective ionic liquid vacuum microwave-assisted method was developed for extraction of the thermo- and oxygen-sensitive glycosides salicin, hyperin and rutin from *Populus* bark due to the strong solvating effects of ionic liquids on plant cell walls. In this study, [C_4_mim]BF_4_ solution was selected as the extracting solution for extraction of the target analytes. After optimization by single factor experiments and response surface methodology, the optimum condition parameters were achieved, which included 1.0 M [C_4_mim]BF_4_, 2 h soaking time, −0.08 MPa vacuum, 20 min microwave irradiation time, 400 W microwave irradiation power and 25 mL/g liquid/solid ratio. Under the optimum conditions, higher extraction yields of salicin (35.53 mg/g), hyperin (1.32 mg/g) and rutin (2.40 mg/g) were obtained. Compared with other extraction methods, the developed method provided higher yields of the three target components after a relatively shorter extraction time (20 min). No obvious degradation of the target analytes was observed under the optimum conditions in performed stability studies and the proposed method had a high reproducibility. Meanwhile, after adsorption and desorption on macroporous D101 resin, the target analytes can be effectively separated from the [C_4_mim]BF_4_ ionic liquid extraction solution and the yields of salicin, hyperin and rutin were 89%, 82% and 84%, respectively. The recovered [C_4_mim]BF_4_ ionic liquid presented a good extraction effect on the three analytes after recycling five times.

## 1. Introduction

*Populus* is a genus of more than 100 species of deciduous plants in the family Salicaceae, These plants, commonly known as poplar, aspen and cottonwood, are native to Europe, Asia and North America [1,2]. The poplars are among the most important boreal broadleaf trees, and are almost all grown as ornamental trees and street trees. *Populus alba × P. berolinensis* is popular for its rapid growth, adaptability, fine grain and pale color. In China, it has been used widely in forests planted for ecological protection, the Three-North Shelterbelt, agroforestry, industrial plantations, near roads and in garden landscaping [3]. Wood from *P. alba × P. berolinensis* is used in civil construction, furniture, and plant fiber materials. These uses produce large quantities of bark as a side product. Consequently, attention has focused on potential uses for *P. alba × P. berolinensis* bark. The bark of *P. alba × P. berolinensis* reportedly contains valuable glycosides, such as salicin and rutin [4,5], and hyperin [6], and extraction of these bioactive components from *P. alba × P. berolinensis* bark would be useful.

Salicin can be used to treat rheumatic fever and subacute bacterial endocarditis [7]. It is also used as a traditional analgesic [8,9]. Salicin is a prodrug that is gradually converted into salicylic acid after absorption, and is antipyretic without causing gastric injury [10]. Rutin shows anti-inflammatory and antioxidant activity [11], can reduce the cytotoxicity of oxidized LDL cholesterol and lower the risk of heart disease [12], inhibit platelet aggregation [13], decrease capillary permeability, and improve circulation [14]. Hyperin is anti-inflammatory [15,16], antioxidant *in vivo* [17,18], antiviral [19], antidepressant [20,21], neuroprotective [22], vascularprotective [23] and hepatoprotective [24]. Because these three natural compounds have many potential applications and economic and environmental value, they are attracting increasing attention.

Conventionally, glycosides are extracted from plant material by heating under reflux (HRE) and Soxhlet extraction. However, the long-term exposure to high temperatures of these methods leads to loss of glycosides because of condensation, degradation, isomerization, or oxidation during extraction. These issues make the method inefficient, require large volumes of toxic organic solvents and result in low recovery of the products. It would therefore be desirable to develop new environmentally friendly methods that can be scaled up for commercial production.

Ionic liquids which are composed of organic cations and inorganic or organic anions are liquid near room temperature. In recent years, ionic liquids have been used as attractive ‘green’ alternatives to conventional volatile organic solvents in various applications, particularly in separation science [25,26]. They have been used for a variety of applications, such as alkaloids [27,28], metal ions [29], flavonoids [30,31], organic acids [32], glycosides [31,33], lignans [34] and proanthocyanidins [35] due to their unique chemical and physical properties, such as negligible vapor pressure, wide liquid range, good stability, tunable viscosity, good miscibility in water and organic solvents, good solubility and extractability for various organic compounds and remarkable advantage of easy to be controlled over conventional solvents [34]. The results of these studies have demonstrated that the use of ionic liquids as alternative solvents to replace traditional organic solvents in extraction is very promising. Heretofore, however, to our best knowledge, how to separate target analytes from ionic liquids has rarely been reported in the literature.

Compared with conventional extraction, MAE uses less solvent and is faster, while providing equivalent or higher extraction yields for the analytes. The effect of microwave is to diffuse the inner components, change the internal microscopic structure and cause the swelling of cells or the breakdown of cell walls when the temperature rises, which enhances mass transfer of the cell contents. Therefore, MAE is attractive for the extraction of active compounds from herbs. However, the operating temperature for MAE in an open vessel is typically close to the boiling point of the solvent, and salicin, hyperin and rutin are thermo- and oxygen-sensitive and rapidly degrade or oxidize in an open system. Therefore, a new method for the extraction of thermo-sensitive compounds is needed.

To overcome the degradation at high operating temperatures in open MAE, vacuum microwave-assisted extraction (VMAE) has been developed [36,37]. Under vacuum, the boiling point of an extraction solvent will be lower than at atmospheric pressure, and the extraction can be performed at lower temperature, which is beneficial for thermo-sensitive compounds. Two major advantages of combining the application of microwave with vacuum techniques are rapid drying due to the ability of microwave to heat solvents instantaneously and homogeneously, and enhanced rate and extent of mass transfer at sub-atmospheric pressure and low temperature. The advantages of VMAE are mainly attributed to the unique extraction mechanism of MAE and the excellent effect of vacuum. In VMAE, because the air in the extraction system is removed, degradation of oxygen-sensitive compounds is avoided or reduced compared to in open MAE [38,39], and this can increase the extraction yield [40].

The objective of this study was to develop an effective and environmentally friendly ionic liquid vacuum microwave-assisted extraction (ILVMAE) method for the extraction of thermo-sensitive and oxygen-sensitive glycosides, salicin, hyperin and rutin from *P. alba × P. berolinensis* bark. The effects of changing the ionic liquid concentration, bark soaking time, microwave irradiation power and time, liquid/solid ratio, and level of vacuum on the extraction yield were evaluated using a factorial design and response surface methodology (RSM) with a Box-Behnken design (BBD). And the proposed method was validated in stability, repeatability, and recovery experiments. Moreover, the separation of target ingredients from ionic liquid extraction solution using macroporous resin was preliminary attempted. The extraction effect of [C_4_mim]BF_4_ after recovery and recycling on the yields of the three target analytes was investigated. The microstructures of unprocessed and processed bark samples were also investigated by scanning electron microscopy (SEM). Hopefully, this work is helpful for the application for the production of salicin, hyperin and rutin from *Populus* bark and other plants.

## 2. Results and Discussion

### 2.1. Screening of Ionic Liquids

The structure of an ionic liquid influences its physicochemical properties, and this might affect the extraction yields of target analytes. According to literature procedures, we screened many types of ionic liquids to select the best one for the extraction based on the extraction yield. The anion and alkyl chain length of the cation were compared. The optimal ionic liquid for extraction was sought and the general trends observed are described below.

#### 2.1.1. Anion Effect

**Figure 1:**
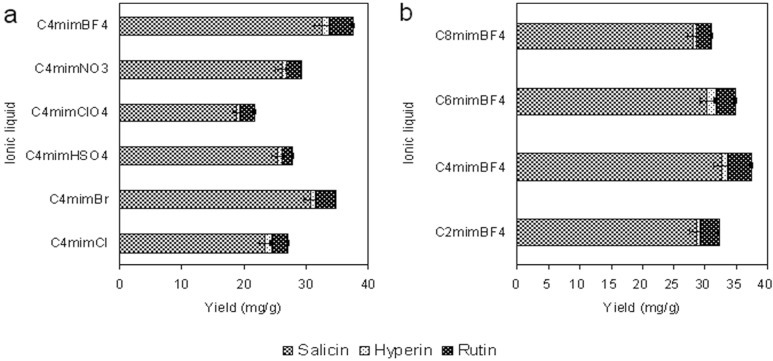
Effect of anion (**a**) and carbon chain length of cation (**b**) on the extraction yields of target analytes. All experiments were performed in triplicate.

The anion identity is considered to strongly influence an ionic liquid’s properties. *N*-Methylimidazolium based ionic liquids with six different anions (single anion: Cl^−^, Br^−^; complex anion: HSO_4_^−^, ClO_4_^−^, NO_3_^−^ and BF_4_^−^) were studied and difference in their extraction yields was readily apparent, as shown in Figure 1a. All of the ionic liquids tested were sufficiently hydrophilic to dissolve in any proportion with water. The results showed that the ionic liquids based on BF_4_^−^ was the most efficient of the ionic liquids tested and BF_4_^−^ was selected for subsequent experiments.

#### 2.1.2. Effect of the Alkyl Chain Length of the Ionic Liquid Cation

As can be seen in Figure 1b, a series of *N*-methylimidazolium cations including C_2_mim^+^, C_4_mim^+^, C_6_mim^+^, and C_8_mim^+^ were evaluated using the same BF_4_^−^ anion. The total extraction yield was found to increase significantly when the alkyl chain length was increased from ethyl to butyl. These can be explained as follows, the solubility of butyl with the three target analytes is more efficient than ethyl. And total extraction yield kept sustained decrease with the change of alkyl chain length from butyl to octyl, this result suggested that steric clash get bigger and bigger, which was similar to previous studies [25,27]. Summing up the above results, C_4_mim^+^ was the best. Having optimized both the anion and cation of the ionic liquid, [C_4_mim]BF_4_ was selected for subsequent extraction parameter optimization studies.

### 2.2. Optimization of Salicin, Hyperin and Rutin Extraction Using a Factorial Design

The univariate method was used to optimize the following parameters: [C_4_mim]BF_4_ concentration, bark soaking time, microwave irradiation power, microwave irradiation time, vacuum, and liquid/solid ratio.

#### 2.2.1. Effect of Concentration

**Figure 2:**
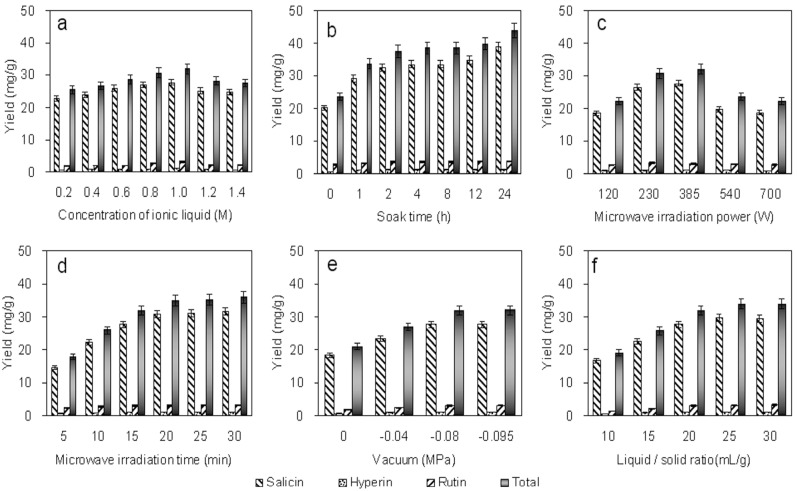
Optimization of salicin, hyperin and rutin extraction using a factorial design. Effects of ionic liquid concentration (**a**), soaking time (**b**), vacuum microwave irradiation power (**c**), vacuum microwave irradiation time (**d**), vacuum drgree (**e**) and liquid/solid ratio (**f**) on the extraction yields of target analytes. All experiments were performed in triplicate.

The extractions were carried out in aqueous solutions of different concentrations (from 0.2 M to 1.4 M). Figure 2a shows that the extraction yields of salicin, rutin and hyperin from *P. alba × P. berolinensis* bark increased as the [C_4_mim]BF_4_ concentration increased. When with the 1.0 M [C_4_mim]BF_4_, the total extraction yield of the three target analytes reached the maximum value. However, when the [C_4_mim]BF_4_ concentration was increased further, the total extraction yield decreased gradually. We conjecture that the high viscosity of the solvent at high ionic liquid concentrations may bring about poor penetration of the solvent into the plant tissue, resulting in decreasing extraction yield. Therefore, 1.0 M [C_4_mim]BF_4_ was selected for subsequent experiments.

#### 2.2.2. Effect of Bark Soaking Time

Experiments were conducted by soaking the bark powers in 1.0 M [C_4_mim]BF_4_ for 0, 1, 2, 4, 8, 12 or 24 h before ionic liquid vacuum microwave-assisted extraction (ILVMAE). These samples were extracted in the microwave oven for 15 min at 385 W. Figure 2b shows the effect of the soaking time on the extraction yields of salicin, hyperin and rutin at room temperature. A substantial increase in the total extraction yield was obtained after soaking the bark. The salicin, hyperin and rutin extraction yields increased significantly when the soaking time was increased from 0 to 2 h. However, longer soaking times did not result in any further improvement in the total extraction yield. The total extraction yield obtained at 2 h (37.49 mg/g) socking time was similar with that obtained when socking time was 4 h, 8 h and 12 h, and could reach 85.36% of that obtained at 24 h (43.92 mg/g). For saving time, thus 2 h was chosen as the optimal soaking time.

#### 2.2.3. Effect of Microwave Irradiation Power

Optimization of the microwave irradiation power used during ILVMAE is very important to ensure efficient extraction. Microwave irradiation powers of 120, 230, 385, 540, and 700 W were investigated. The extraction yields of salicin, hyperin and rutin increased as the microwave irradiation power increased from 100 W to 385 W, and decreased as the microwave irradiation power increased from 385 W to 700 W (Figure 2c). The microwave irradiation time was constant throughout this experiment at 15 min. A 230–540 W microwave irradiation power was selected for subsequent experiments.

#### 2.2.4. Effect of Microwave Irradiation Time

The microwave irradiation time is important in determining the extraction yields. The ILVMAE power was constant throughout this experiment at 385 W. Figure 2d shows that as the irradiation time increased from 5 to 20 min, the extraction yields of salicin, hyperin and rutin increased dramatically. When the irradiation time increased from 20 to 30 min, only slight improvements were observed. Therefore, a 10–20 min irradiation time was selected for subsequent experiments.

#### 2.2.5. Effect of the Vacuum

The vacuum was set at four different levels (0, −0.04, −0.08 and −0.095 MPa). Figure 2e shows the effect of the vacuum on the extraction yields of salicin, hyperin and rutin at room temperature. The total extraction yield increased drastically as the pressure was decreased from 0 MPa to −0.08 MPa. However, no obvious improvement of the extraction yield was observed when pressure decreased below −0.08 Mpa. Therefore, a vacuum of −0.08 MPa was used in subsequent experiments.

#### 2.2.6. Effect of the Liquid/Solid Ratio

The liquid/solid ratio is an important factor because large solvent volumes could make the procedure difficult and lead to unnecessary solvent waste. By contrast, small solvent volumes may lead to incomplete extraction. A series of experiments were carried out with different liquid/solid ratios (10, 15, 20, 25 and 30, mL/g). Figure 2f shows that the total extraction yield increased as the liquid/solid ratio increased up to 20 mL/g. However, higher liquid/solid ratios did not significantly improve the total extraction yield. Thus, a liquid/solid ratio between 15 and 25 mL/g was used in subsequent experiments.

### 2.3. Optimization of Salicin, Hyperin and Rutin Extraction Using Response Surface Methodology (RSM)

To further study the interactions between the factors, we optimized the microwave irradiation time, microwave irradiation power and liquid/solid ratio by RSM. From Table 1, a model *F*-value of 36.10 indicated that the model was significant, and there was only a <0.01% chance that a model *F*-value of this size could occur due to statistical noise. Values of “Probability > *F*” less than 0.0500 indicated the model terms were significant. In this case, *A*, *B*, *C, AC* and *B^2^* were significant model terms. Values of “Probability > *F*” greater than 0.1000 indicated that the model terms were not significant. A “Lack of fit F-value” of 2.87 implied that the “Lack of fit” was not significant. The probability for the occurrence of this “Lack of fit *F*-value” was 16.74% and can be treated as statistical noise. The “Predicted *R*^2^” of 0.7585 is in reasonable agreement with the “Adjust *R*^2^” of 0.9517. “Adequacy precision” measures the signal to noise ratio. A ratio greater than 4 is desirable. The ratio of 24.278 indicates an adequate signal. This model can be used to navigate the design space. The final total extraction yield of salicin, hyperin and rutin (*Y*) was given by the following equation:

*Y* = 22.515 − 0.577*A* + 0.103*B* − 0.695*C* + 0.048*AC* (1)

**Figure 3:**
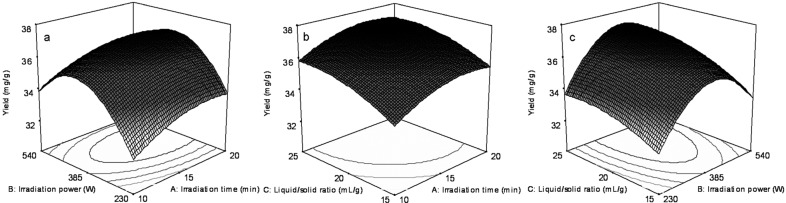
Optimization of salicin, hyperin and rutin extraction using BBD. Response surface plots showing the effects of variables on total extraction yield of target analytes. (**a**) Interaction of vacuum microwave irradiation time and power; (**b**) Interaction of vacuum microwave irradiation time and liquid/solid ratio; (**c**) Interaction of vacuum microwave irradiation power and liquid/solid ratio. All experiments were performed in triplicate.

The response surfaces for the effect of independent variables on the total extraction yield of salicin, hyperin and rutin are shown in Figure 3. Figure 3a shows the three-dimensional response surface plots at various microwave irradiation times (*A*) and microwave irradiation powers (*B*) with a fixed liquid/solid ratio (0 level). The microwave irradiation power (*B*) could be optimized to obtain the highest total extraction yield of salicin, hyperin and rutin. Powers lower or higher than this optimum value decreased the total extraction yield. The productivity was maximized when the microwave irradiation power (*B*) was 385 (W). Figure 3b shows the three-dimensional response surface plots at various microwave irradiation times (*A*) and liquid/solid ratios (*C*) with a fixed microwave irradiation power (0 level). The total extraction yield of salicin, hyperin and rutin increased slowly as the liquid/solid ratio increased (*C*) and the microwave irradiation time increased (*A*). The total extraction yield then remained constant with further increases in the liquid/solid ratio (*C*) or microwave irradiation time (A). Figure 3c shows the three-dimensional response surface plots at various microwave irradiation powers (*B*) and liquid/solid ratios (*C*) with a fixed microwave irradiation time (0 level). The microwave irradiation power (*B*) affected the total extraction yield. The total extraction yield of salicin, hyperin and rutin significantly increased and then decreased as the microwave irradiation power (*B*) was increased. The maximum total extraction yield was obtained with a microwave irradiation power (*B*) of 385 (W). The conditions for point prediction by software were as follows: 20 min microwave irradiation time, 404 W microwave irradiation power, and 25 liquid/solid ratio (mL/g). Under the conditions of point prediction, the total extraction yield reached 40.84 mg/g.

**Table 1:** Experimental design matrix to screen for variables that determine the total extraction yield of salicin, hyperin and rutin from *Poplus alba* × *P. berolinensis* bark and ANOVA results ^a^.

Run	BBD Experiments				ANOVA									
	A (min)	B(W)	C (mL/g)	Y (mg/g)	Source		Sum of Squares		Degree of Freedom		Mean Square		*F*-value	*p*-value
1	20	385	25	41.22	Model^b^		81.45		9		9.05		36.10	< 0.0001 ^c^
2	10	385	25	35.83	*A*		12.98		1		12.98		51.63	0.0002 ^c^
3	10	230	20	31.43	*B*		4.21		1		4.21		16.73	0.0046 ^c^
4	15	385	20	37.12	*C*		17.02		1		17.02		67.71	<0.0001 ^c^
5	15	385	20	36.21	*AB*		0.13		1		0.13		0.50	0.5018
6	15	230	15	32.24	*AC*		5.81		1		5.81		23.10	0.0020 ^c^
7	15	230	25	34.34	*BC*		0.16		1		0.16		0.62	0.4567
8	15	540	25	35.70	*A*^2^		0.01		1		0.01		0.02	0.8855
9	10	540	20	33.72	*B*^2^		40.96		1		40.96		162.93	< 0.0001 ^c^
10	10	385	15	34.90	*C*^2^		0.05		1		0.05		0.18	0.6846
11	15	540	15	32.81	Residual		1.76		7		0.25			
12	15	385	20	37.10	Lack of fit		1.20		3		0.40		2.87	0.1674
13	15	385	20	36.67	Pure error		0.56		4		0.14			
14	20	385	15	35.47	Corrected total		83.21		16					
15	20	540	20	35.48	Credibility analysis of the regression equations									
16	15	385	20	36.84	Index mark	Standard deviation		Mean	Coefficient of variation %	Press	R^2^	Adjust R^2^	Predicted R^2^	Adequacy precision
17	20	230	20	33.90	*Y*	0.50		35.35	1.42	20.10	0.9789	0.9517	0.7585	24.278

^a^ The results were obtained with Design Expert 7.0 software [41]. ^b^ A is the vacuum microwave irradiation time (min), B is the vacuum microwave irradiation power (W), C is the liquid–solid ratio (mL/g), and Y is the total extraction yield (mg/g). ^c^ Significant at *p* < 0.05.

### 2.4. Verification Test under Optimum Condition

The verification test was done three times under the conditions of point prediction by RSM (1.0 M [C_4_mim]BF_4_, 2 h soak, −0.08 MPa vacuum, 20 min microwave irradiation at 400 W, and 25 mL/g liquid/solid ratio). The actual total extraction yield was 39.25 mg/g (for salicin was 35.53 mg/g, for hyperin was 1.32 mg/g and for rutin was 2.40 mg/g) with an error of 1.59 mg/g. The same bark sample was extracted under the optimum parameters derived for several times until no obvious improvement of the yields was obtained, and the extraction yields of the three analytes determined were taken as the real quantities of products in raw material. After extraction under the optimum conditions for one time, the extract was determined by HPLC, namely the quantities of products in extract. The recovery yields of the salicin, hyperin and rutin were 92.32%, 95.17% and 93.68%, respectively, which means that most of the salicin, hyperin and rutin were extracted from bark samples by the developed ILVMAE method:

Yield (%) = (quantity of product in extract/quantity of product in raw material) × 100
(2)

### 2.5. Method Validation

#### 2.5.1. Stability Studies of Standard Stock Solutions of Salicin, Hyperin and Rutin in Methanol

Intra- and inter-day stability studies were performed based on the standard solutions at concentrations of salicin of 14.62, 7.31 and 1.462 mg/mL, hyperin of 2.54, 1.27 and 0.254 mg/mL, rutin of 2.66, 1.33 and 0.266 mg/mL, respectively. Each experiment was repeated five times. The results of intra-day and inter-day tests are presented in Table 2. Stock solutions of salicin, hyperin and rutin in methanol was found to be stable for at least 4 h when stored at 18–20 °C and the final concentrations were 99.21%–100.39% of the initial values. The inter-day result indicated that standard solutions of salicin, hyperin and rutin were stable in methanol for at least 5 d when the samples were kept at 4–8 °C and the final concentrations were 99.21%–100.07% of the initial values.

**Table 2:** Stability studies of standard stock solutions of salicin, hyperin and rutin standards in methanol (*n* = 5).

Indicators	Number	Compounds		
		Salicin	Hyperin	Rutin
Standard solution	1	14.62	2.54	2.66
	2	7.31	1.27	1.33
	3	1.462	0.254	0.266
Recovered concentration after 4 h (mg/mL)	1	14.60	2.55	2.65
	2	7.32	1.26	1.32
	3	1.462	0.253	0.267
RSD% (*n* = 5)	1	1.11	0.97	0.96
	2	0.98	0.98	0.98
	3	0.98	0.93	1.01
Average recovery of intra-day (%)	1	99.86	100.39	99.62
	2	100.14	99.21	99.25
	3	100.00	99.61	100.38
Recovered concentration after 5d (mg/mL)	1	14.61	2.54	2.66
	2	7.31	1.26	1.33
	3	1.463	0.254	0.265
RSD% (*n* = 5)	1	1.05	0.99	0.97
	2	0.97	0.97	0.98
	3	1.00	0.97	0.99
Average recovery of inter-day (%)	1	99.93	100.00	100.00
	2	100.00	99.21	100.00
	3	100.07	100.00	99.62

#### 2.5.2. Stability Studies of Salicin, Hyperin and Rutin Standards under the ILVMAE Conditions

The stability of the glucosides under the optimum ILVMAE conditions was assessed by extracting salicin, hyperin and rutin standards with 1.0 M [C_4_mim]BF_4_ using a 2 h soak, −0.08 MPa vacuum, 400 W microwave power, 20 min irradiation time, and liquid/solid ratio of 25 mL/g. Each experiment was repeated for three times. Recovery of the glucosides was assumed to indicate their stabilities under these extraction conditions (Table 3).

**Table 3:** Stability studies of salicin, hyperin and rutin standards in 1M C_4_mimBF_4_ extraction solution under the optimum ILVMAE conditions (n = 3).

Compounds	Initial Concentration (mg/mL)	Intra-day			Inter-day		
		Recovered Concentration (mg/mL)	RSD%	Average Recovery (%)	Recovered Concentration (mg/mL)	RSD%	Average Recovery (%)
Salicin	1.46	1.43	0.98	97.9	1.42	0.99	97.1
Hyperin	0.25	0.26	1.02	102.0	0.24	0.95	96.8
Rutin	0.27	0.26	0.97	97.1	0.26	0.97	94.8

The average recovery obtained from intra-dey assays varied from 97.1% to 102.0% with no change in retention time observed for the glucosides. The inter-day result indicated that the average recoveries of salicin, hyperin and rutin were 97.1%, 96.8% and 94.8% after stored in 1.0 M [C_4_mim]BF_4_ for some days. Thus salicin, hyperin and rutin were stable in 1.0 M [C_4_mim]BF_4_ extract solution under the selected optimum conditions.

#### 2.5.3. Recovery

Under the optimized conditions detailed above, bark samples that were spiked with salicin, hyperin and rutin at three concentrations were extracted and each process was repeated for three times. The results are shown in Table 4. The recoveries of salicin, hyperin and rutin from *P. alba* × *P. berolinensis* bark were 101.0%, 100.2% and 99.6%, respectively.

**Table 4:** Recovery of salicin, hyperin and rutin from dried bark samples of *Poplus alba* × *P. berolinensis* (*n* = 3).

Sample	Spiked Mean Mass (mg)			Mean Mass in Sample (before Addition) (mg)			Detected Mean Mass (after Addition) (mg)			Recovery (%)		
	Salicin	Hyperin	Rutin	Salicin	Hyperin	Rutin	Salicin	Hyperin	Rutin	Salicin	Hyperin	Rutin
1	0.5	0.05	0.1	1.12	0.13	0.22	1.56	0.18	0.31	96.3	100.0	96.9
2	1.0	0.1	0.2	1.12	0.13	0.22	2.17	0.24	0.42	102.4	104.3	100.0
3	1.5	0.15	0.3	1.12	0.13	0.22	2.73	0.27	0.53	104.2	96.4	101.9
Average										101.0	100.2	99.6

#### 2.5.4. Repeatability

To determine the repeatability of the novel extraction method, bark samples were processed on five consecutive days under the optimum extraction conditions. Each experiment was repeated for five times. The result obtained from experiments conducted on different days was shown in Table 5, from which we can see that the extraction yields of salicin, hyperin and rutin with calculated relative standard deviations were 35.52% ± 3.6%, 1.32% ± 5.2% and 2.39% ± 4.6%, respectively. This shows that the proposed ILVMAE method has an acceptable level of repeatability.

**Table 5:** Repeatability studies under the following ILVMAE conditions: 2 h soak, 0.08 MPa vacuum, 400 W microwave irradiation power, 20 min microwave irradiation time, 25:1 liquid/solid ratio (mL/g), 1M C_4_mimBF_4_ as the extraction solvent (n = 5).

Repeat Number	Yield (mg/g)		
	Salicin	Hyperin	Rutin
1 (1st day)	36.551	1.388	2.359
2 (1st day)	33.922	1.296	2.326
3 (2nd day)	35.663	1.341	2.342
4 (3rd day)	34.548	1.363	2.337
5 (5th day)	36.916	1.212	2.586
Average	35.52	1.32	2.39
RSD (%)	3.6	5.2	4.6

The results suggested that salicin, hyperin and rutin were stable in 1.0 M [C_4_mim]BF_4_ and extracts under the optimized conditions. Because of its good repeatability and precision, the proposed ILVMAE method is promising for the extraction and separation of herbal products.

### 2.6. Comparison of ILVMAE with Other Methods

The extraction yields obtained of salicin, hyperin and rutin from *P. alba* × *P. berolinensis* bark with ILVMAE, ionic liquid microwave-assisted extraction (ILMAE) and ionic liquid based heat reflux extraction (ILHRE) were compared. As shown in Table 6, the extraction yields of salicin, hyperin and rutin were much higher with ILVMAE than with ILHRE. The total extraction yields for ILVMAE, ILMAE and ILHRE were 39.25 ± 1.59 mg/g, 32.51 ± 1.32 mg/g, and 28.27 ± 1.18 mg/g, respectively. The ILVMAE extraction was also faster, with the ILHRE taking 120 min and the ILVMAE extraction only 20 min. The comparison of ILVMAE to ILMAE showed the vacuum was important for increasing the extraction yields. Therefore, ILVMAE is an efficient method for the extraction of salicin, hyperin and rutin from *P. alba* × *P. berolinensis* bark.

**Table 6:** Comparison of ILVMAE with other extraction methods, mean ± S.D. (n = 3).

Number	Method	Extraction Time (min)	Extraction Yield ± SD (mg/g)			
			Salicin	Hyperin	Rutin	Total
1	ILVMAE	15	35.53 ± 1.40	1.32 ± 0.04	2.40 ± 0.15	39.25 ± 1.59
2	ILMAE	15	28.43 ± 1.14	0.96 ± 0.04	3.12 ± 0.14	32.51 ± 1.32
3	ILHRE	120	24.49 ± 1.03	0.92 ± 0.03	2.86 ± 0.12	28.27 ± 1.18

### 2.7. Separation of Target Analytes from Ionic Liquid Extraction Solution

**Figure 4:**
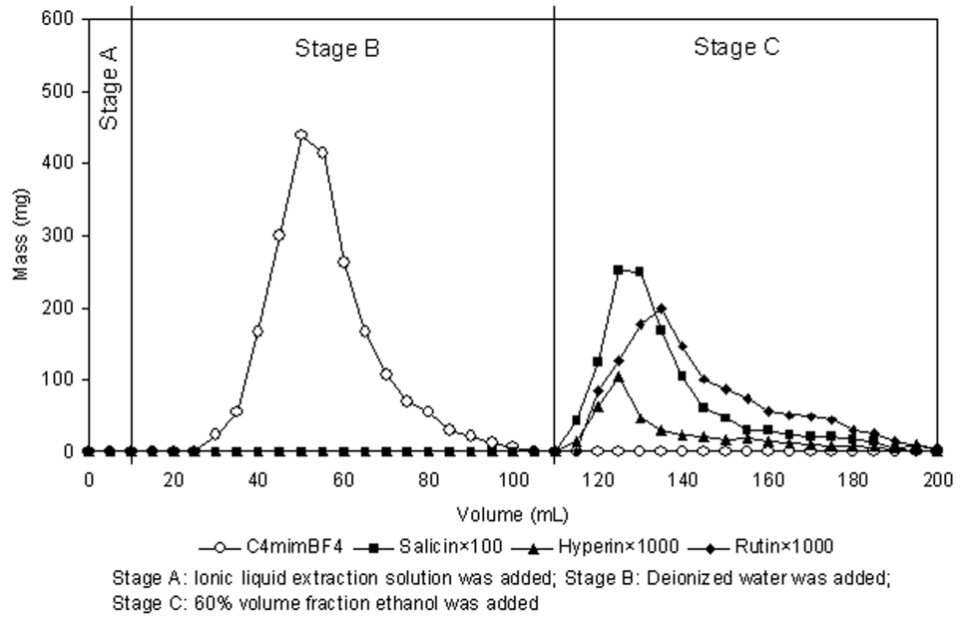
Dynamic desorption curves of ionic liquid and target analytes on a column packed with D101 macroporous resin.

Dynamic adsorption and desorption process using a wet-packed fixed-bed column separator was performed according to a previous study [42]. The initial concentrations of salicin, hyperin and rutin in this test were 1.35, 0.05 and 0.15 mg/mL, and the flow rate in this test was 1BV/h for ionic liquid extraction solution, 2BV/h for water washing process and 60% volume fraction ethanol desorption process, respectively. The results, which are shown in Figure 4, indicated that most of ionic liquid in the extraction solution can be flowed out in the water washing process and the cumulative outflow of [C_4_mim]BF_4_ was more than 93% through HPLC detection. The effectively separation of three target analytes from ionic liquid extraction solution can be achieved through absorption of D101 resin and desorption of 60% volume fraction ethanol, and their yields were more than 89%, 82% and 84%, respectively.

### 2.8. Recovery and Recycling of Ionic Liquid

**Figure 5:**
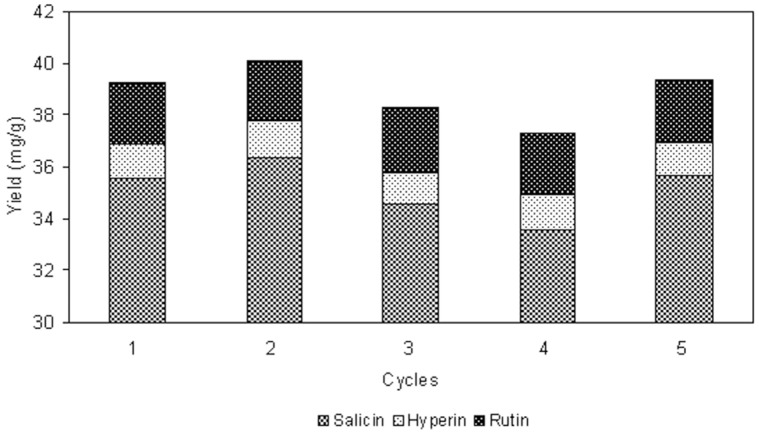
The performance of the recovered [C_4_mim]BF_4_ on the yields of salicin, hyperin and rutin.

As can be seen from Figure 4, 20–110 mL of effluent solution was collected and evaporated under 90 °C for recovery of water in −0.09 MPa of pressure, by which the sticky [C_4_mim]BF_4_ was obtained. Then the recovered [C_4_mim]BF_4_ could thus be reused without further purification. An important advantage of the ionic liquid represents its recyclable usage. Consequently, the recycling usage of [C_4_mim]BF_4_ was studied at 1.0 M [C_4_mim]BF_4_ using a 2 h soak, −0.08 MPa vacuum, 400 W microwave power, 20 min irradiation time, and liquid/solid ratio of 25 mL/g, each experiment was repeated three times. No significant loss in the extraction yields of three target analytes was observed when the [C_4_mim]BF_4_ was repeatedly used for the extraction process. As shown in Figure 5, after one‑run [C_4_mim]BF_4_ treatment, the total yield of three target analytes was 40.08 mg/g, comparing with 39.25 mg/g yield by [C_4_mim]BF_4_ initial use. The [C_4_mim]BF_4_ that had been repeatedly used five times still had efficient extraction, the total yield of three target analytes could reach 39.38 mg/g. Therefore, the method for recovery and recycling of ionic liquid was effective and can be used for recycling usage of ionic liquid in other experiments.

### 2.9. Structural Changes after Extraction

**Figure 6:**
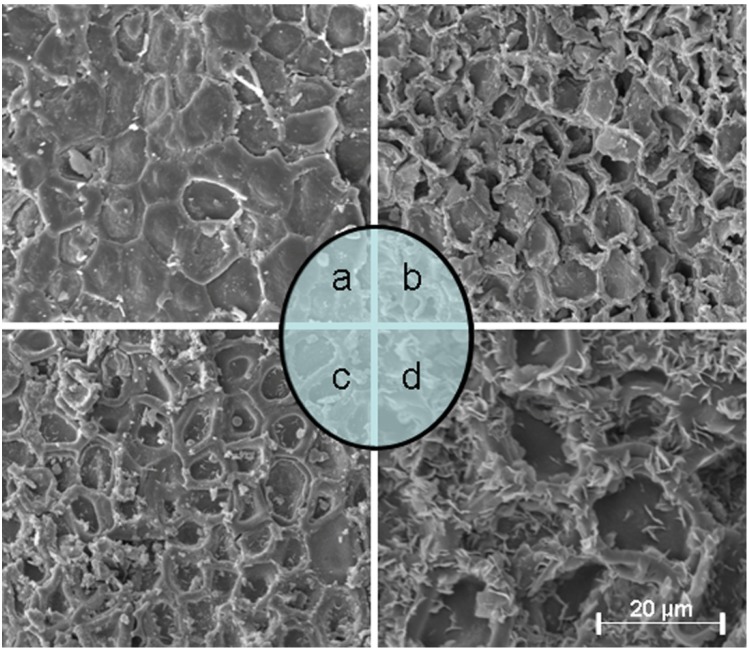
Scanning electron microscopy images of bark of *Poplus alba* × *P. berolinensis*: (**a**) untreated sample; (**b**) ILVMAE; (**c**) ILMAE; (**d**) ILHRE.

The various extraction methods produced different physical changes in *P. alba* × *P. berolinensis* bark. To investigate these physical changes, the bark samples were analyzed by scanning electron microscopy SEM. Figure 6 shows the micrographs of the bark samples before and after the different extraction methods. Figure 6a shows the micrograph of untreated bark and Figure 6b–d shows the micrographs of bark samples treated by ILVMAE (20 min), ILMAE (20 min) and ILHRE (2 h). Numerous unbroken cells were observed. After ILHRE, some of the cell walls were ruptured, but many remained unbroken. Consequently, in this method, the solvent has to transfer into the sample to extract the compounds, which leads to the longer extraction time. The minimal changes in microstructure could be attributed to the heat transfer during ILHRE occurring by conduction and convection only [43]. By contrast, during ILVMAE and ILMAE, the heat transfer is by radiation, conduction, and convection [44]. After ILVMAE and ILMAE, most of the cells and cell walls were broken. This would expose the target analytes to the extraction solution, and reduce the extraction time. The severe thermal stresses and localized high pressures in microwave heating could contribute to their ruptures.

## 3. Experimental Section

### 3.1. Materials and Chemicals

*P. alba* × *P. berolinensis* bark was provided by the Maoershan Experimental Forest Farm of Northeast Forestry University (Harbin, Heilongjiang, China), and authenticated by Prof. Shaoquan Nie from State Engineering Laboratory for Bio-Resource Eco-Utilization (Northeast Forestry University). The bark was dried at room temperature for a month, and then pulverized using a blender and sieved (60–80 mesh) before extraction. The pulverized samples were stored in closed desiccators at 4 °C until use. The same batch of sample was used in all experiments. Reference samples of salicin, hyperin and rutin were purchased from the National Institute for the Control of Pharmaceutical and Biological Products (Beijing, China). Methanol used for HPLC analysis was of chromatographic grade and purchased from J&K Chemical Ltd. (Beijing, China). All the other reagents were obtained from Beijing Chemical Reagents Co. (Beijing, China) and were of analytical grade. All ionic liquids ([C_4_mim]Cl, [C_4_mim]Br, [C_4_mim]BF_4_, [C_4_mim]NO_3_, [C_4_mim]HSO_4_, [C_4_mim]ClO_4_, [C_2_mim]BF_4_, [C_6_mim]BF_4_, [C_8_mim]BF_4_, where C_2_ = 1-ethyl, C_4_ = 1-butyl, C_6_ = 1-hexyl, C_8_ = 1-octyl, and mim = 3-methylimidazolium) were bought from Shanghai Cheng Jie Chemical Co. LTD. (Shanghai, China) and used without further purification. Deionized water purified by a Milli-Q water purification system (Millipore, Bedford, MA, USA) was used for preparing and diluting all solutions. All of the solvents for HPLC were filtered through a 0.45 µm microporous membrane (Guangfu Fine Chemicals Research Institute, Tianjin, China) and degassed by ultrasonication before use.

D101 macroporous resin (surface area 500–550 m^2^/g, average pore diameter 9–10 nm, particle diameter 0.30–1.25 mm, crosslinked polystyrene, non-polar) was purchased from Anhui Sanxing Resin Technology Co., Ltd. (Guzhen, Anhui, China). In order to remove the monomers and porogenic agents trapped inside the pores of macroporous resin during synthesis process, the adsorbent bead was pretreated with the following procedure: first, the resin was soaked in ethanol for 24 h, and then washed with deionized water by circumfluence until there is no residue of ethanol [45]. The treated resin was stored in a desiccator with deionized water in order to maintain constant moisture content. Prior to use, the resin was wet with ethanol again and then thoroughly replaced with deionized water [46]. The moisture content of the tested D101 macroporous resin was 66.65%.

### 3.2. Apparatus

**Figure 7:**
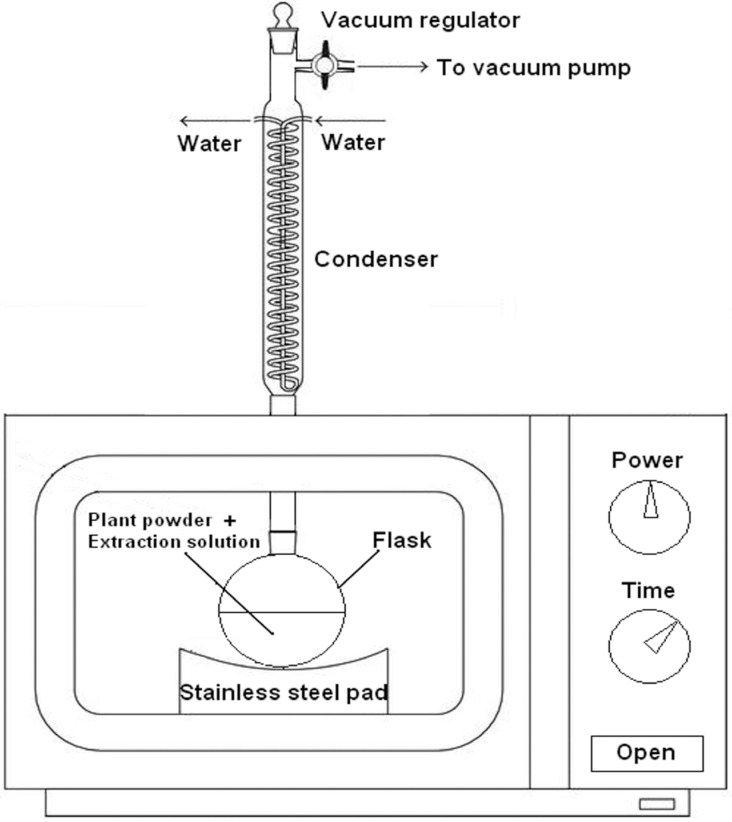
Schematic diagram of the experimental apparatus.

A domestic WP700 microwave-assisted extraction unit (irradiation frequency 2.45 GHz, maximum output power 700 W and with continuously adjustable power; Glanz Electrical Appliance Industrial Co., Ltd., Guangzhou, Guangdong, China) was used for the extraction. The dimensions of the interior cavity of the oven were 215 mm × 350 mm × 330 mm. The microwave was modified in our laboratory with the addition of a water condenser coated with polytetrafluoroethene to prevent microwave leakage. The whole system was run under vacuum. The vacuum in the system was created with a vacuum pump (SHB-IV, Zhengzhou Greatwall Scientific Industrial and Trade Co., Ltd., Zhengzhou, Henan, China). The vacuum pump was fitted between the condenser and the flask that was used to collect the crude extract. As shown in Figure 7, the schematic diagram of the VMAE equipment, some parameters which influence the extraction process, such as the vacuum, microwave irradiation power and time, can be set on the equipment.

### 3.3. HPLC Analysis and Quantification

#### 3.3.1. Preparation of Standard Solutions of Salicin, Hyperin and Rutin

Standard stock solutions of salicin (14.62 mg/mL), hyperin (2.54 mg/mL) and rutin (2.66 mg/mL) were prepared in methanol. The standard stock solutions were stored at 4 °C, and diluted with methanol to the required concentration before direct analysis by HPLC.

#### 3.3.2. Stability Test of Standard Mixtures

Standard mixtures of salicin (14.62 mg/mL), hyperin (2.54 mg/mL) and rutin (2.66 mg/mL) were prepared in 25 mL volumetric flasks using 1ml glass bulb pipettes. For HPLC determination, the mixtures were diluted with methanol. Standard mixtures were stored at room temperature (approximately 18–20 °C) for 4 h in volumetric flask. The stability of the sample at room temperature was evaluated by comparing the assay results of the standard mixtures with that of the freshly thawed standard mixtures. Long-term stability was studied by assaying samples that had been stored at 4–8 °C for a certain period of time (5 days).

#### 3.3.3. HPLC analytical Conditions

The chromatographic system (Waters, Milford, MA, USA) was equipped with Millennium 32 software, a 717 plus autosampler, 1525 pump, 717 automatic column temperature control box and 2487 UV detector. Chromatographic separation was performed on a Kromasil-C_18_ reversed-phase column (AkzoNobel Pulp and Performance Chemicals AB Separation Products, Bohus, Sweden; 4.6 mm × 250 mm, 5 µm).

**Figure 8:**
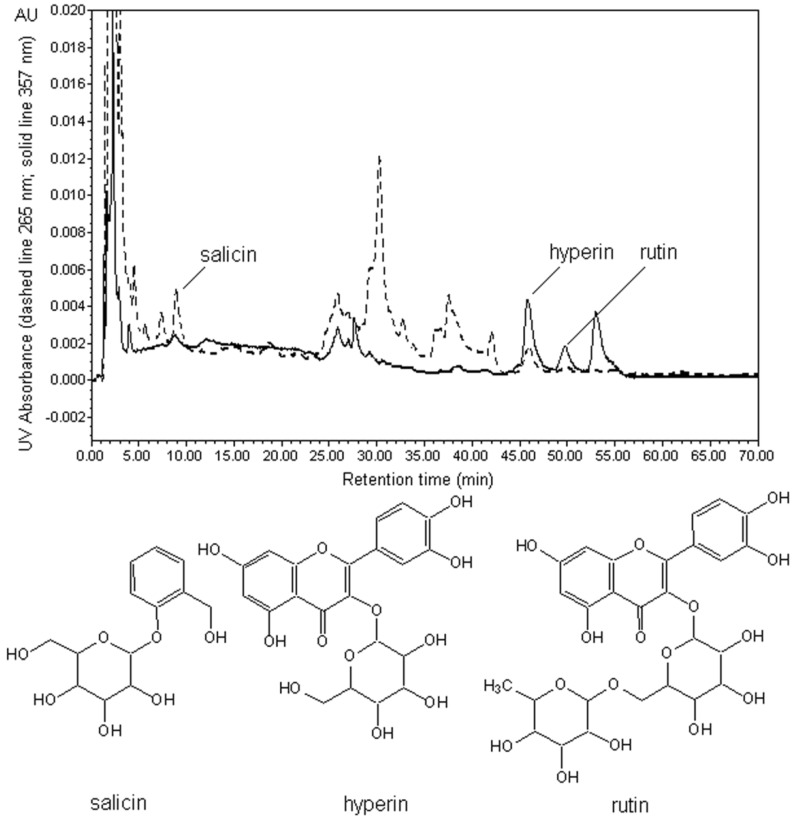
HPLC profile of target analytes in an extract obtained using 1 M C_4_mimBF_4_ as extraction solvent.

The standard solutions of salicin, hyperin and rutin and the extracts were filtered through 0.45 µm membranes before HPLC analysis. The mobile phase used for separation was methanol (A) and deionized water (B). The following gradient elution program was used for separation: 0–15 min, 15% A; 15–18 min, 15%–32% A; 18–68 min, 32% A; and 68–70 min, 32%–15% A. The mobile phase flow rate was 1.5 mL/min, the injection volume was 10 μL, the column temperature was 25 °C and the run time was 70 min. The wavelength used for detection of salicin was 265 nm, and 357 nm was used for hyperin and rutin. Salicin, hyperin and rutin were baseline separated under these conditions. The retention times of salicin, hyperin and rutin were 10.0, 45.1 and 47.8 min, respectively. The chromatographic peaks of the analytes were confirmed by comparing their retention time with reference standards. The results are shown in Figure 8. No influence attributable to the ionic liquids used were observed on peak resolution, elution order or elution time. The linear equations for the calibration curves for salicin, hyperin and rutin were *Y*_salicin_ = 182369*X* – 11025 (*R*^2^ = 0.9999, n = 7), *Y*_hyperin_ = 1897587*X* + 123321 (*R*^2^ = 0.9997, n = 7), and *Y*_rutin_ = 3453739*X* + 83323 (*R*^2^ = 0.9997, n = 7), respectively. The calibration curves showed good linearity for salicin between 0.277 and 14.62 mg/mL, hyperin between 0.00124 and 2.54 mg/mL, and rutin between 0.0026 and 2.66 mg/mL.

For HPLC analysis of [C_4_mim]BF_4_, acetonitrile–1% volume fraction of acetic acid (20:80, v/v) is used as the mobile phase with 1.0 mL/min flow rate, 10 µL injection volume, and 25 °C column temperature. The absorbance was measured at a wavelength of 210 nm, the retention time is 6.8 min, and the corresponding calibration curve is *Y*_IL_ = 3124560*X* + 13725 (*R*^2^ = 0.9998, n = 7). A good linearity was found in the range of 0.0125–5.0 mg/mL.

### 3.4. Ionic Liquid Vacuum Microwave-Assisted Extraction (ILVMAE)

A 0.5 g sample was weighed accurately and into a round-bottom flask containing the extraction solvent. The flask was placed in the microwave oven and connected to a condenser. The air in the vessel was removed until the required vacuum was obtained. Then ILVMAE was performed at a certain temperature. Before the experiment, the sample was mixed with ionic liquid solution and macerated to enhance its ability to absorb microwave energy. The anion, cation, ionic liquid concentration, socking time, microwave irradiation power and time, liquid/solid ratio, and level of vacuum were systematically varied to optimize the extraction yields. After each extraction, the extract was cooled to room temperature rapidly and filtered through a 0.45 μm membrane (Guangfu Fine Chemicals Research Institute, Tianjin, China) for subsequent HPLC analysis. The extraction yields of target analytes were determined as follows:

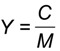
(3)
where *Y* is extraction yield (mg/g); *C* is mean mass of salicin, hyperin or rutin in samples (mg); *M* is mean mass of samples (g). The mean mass of salicin, hyperin and rutin in samples was determined by HPLC analysis of three samples, respectively. The mean mass of the samples was the average mass of three samples before extraction.

### 3.5. Optimization of ILVMAE by RSM

To further study the interaction between the factors, we optimize the operating condition by Box-Behnken design with three factors applied using Design-Expert 7.0 software [41] without any blocking. The boundaries of the factors were 10–20 min for the microwave irradiation time, 230–540 W for the microwave irradiation power, and 15–25 for liquid/solid ratio (mL/g). Specific conditions for each experiment are shown in Table 1.

### 3.6. Stability and Repeatability of ILVMAE

The stability test was performed with salicin, hyperin and rutin standards dissolved in 1.0 M [C_4_mim]BF_4_ and extracted under the optimum conditions (2 h soak time, 0.08 MPa vacuum, 400 W microwave irradiation power, 20 min microwave irradiation time, liquid/solid ratio of 25:1 mL/g). For intra-day assays, the extract was determined once every hour on the same day. The inter-day experiments were performed by determining the extract once daily within the next five days. The recoveries of salicin, hyperin and rutin were taken as indicators of the stabilities of salicin, hyperin and rutin under these extraction conditions. To determine the reproducibility of this extraction method, bark samples were processed on five consecutive days under the optimum extraction conditions.

### 3.7. Comparison of ILVMAE with Reference Extraction Methods

ILMAE and ILHRE were selected as reference extraction methods for the three target analytes. The extraction experiments were operated under the optimized conditions. For ILMAE, 0.5 g of a bark sample was accurately weighed into a round-bottom flask, and 12.5 mL of 1.0 M [C_4_mim]BF_4_ was added. The flask was placed into the microwave oven and irradiated at 400 W for 20 min. The experiment was carried out at atmospheric pressure. For ILHRE, 0.5 g of bark sample was accurately weighed into a round-bottom flask, and 12.5 mL of 1.0 M [C_4_mim]BF_4_ was added. The flask was placed on a heating mantle, connected to a reflux condenser, and heated under reflux for 2 h at 700 W.

### 3.8. Separation of Salicin, Hyperin and Rutin from Ionic Liquid Extraction Solution

The experiment of separation of target analytes from ionic liquid extraction solution was carried out in a glass column (12 mm × 500 mm) (Tianjin Tianbo Glass Instrument Co., Ltd., Tianjin, China) wet‑packed with 5 g (dry weight basis) D101 macroporous resin [47,48,49]. The bed volume (BV) and the length of the resin were 25 mL and 10 cm, respectively. A 100 mL ionic liquid extraction solution was flowed downward and through the glass column at 1 BV/h, and the target analytes concentration was monitored by HPLC analysis of the effluent liquid collected at 5 mL intervals. The adsorbate-laden columns were first washed with 4 BV deionized water at flow rate of 2 BV/h and then with 60% volume fraction of ethanol at flow rate of 2 BV/h. The target analytes concentrations in the effluent solution were determined by HPLC analysis of the effluent liquid collected at 5 mL intervals. The effluent solution were concentrated and dried under vacuum before further analyses and the recoveries of the target analytes were calculated.

### 3.9. SEM

The microstructures of unprocessed and processed bark samples were determined by SEM (Quanta 200, FEI, Hillsboro, OR, USA). To provide electrical conductivity, the sample surface was sputter coated with a thin layer of gold (5–10 nm; 10 mA; 30 s) using an SCD 005 sputter coater (KYKY SBC-12, Beijing, China) at room temperature, as used in literature [50].

### 3.10. Statistical Analysis

One-way ANOVA was used to determine if differences in the extraction yields were significant. The results of HPLC analysis are expressed as means ± S.D.

## 4. Conclusions

An efficient and environmentally friendly ILVMAE method was developed for the extraction of three glucosides, salicin, hyperin and rutin, from *P. alba* × *P. Berolinensis* bark. Under the optimized conditions, satisfactory extraction yields for the three glucosides were obtained. Compared to reference extraction techniques, the developed method was more efficiency and much shorter. The extraction of salicin, hyperin and rutin was readily and efficiently achieved using ILVMAE. Moreover, the salicin, hyperin and rutin can be effectively separated from [C_4_mim]BF_4_ ionic liquid by macroporous resin adsorption and desorption process. After recovery and recycling for five times, the [C_4_mim]BF_4_ still had acceptable extraction yield on the three target analytes, which was significant for saving of ionic liquid and cost.
